# Leveraging the effector independent nature of motor imagery when it is paired with physical practice

**DOI:** 10.1038/s41598-020-78120-9

**Published:** 2020-12-07

**Authors:** Sarah N. Kraeutner, Jennifer L. McArthur, Paul H. Kraeutner, David A. Westwood, Shaun G. Boe

**Affiliations:** 1 Brain Behaviour Laboratory, University of British Columbia, Vancouver, BC V6T1Z3 Canada; 2 Department of Physical Therapy, University of British Columbia, Vancouver, BC V6T1Z3 Canada; 3 Laboratory for Brain Recovery and Function, Dalhousie University, Halifax, NS B3H4R1 Canada; 4 Department of Psychology and Neuroscience, Dalhousie University, Halifax, NS B3H4R2 Canada; 5 School of Health and Human Performance, Dalhousie University, Halifax, NS B3H4R2 Canada; 6 School of Physiotherapy, Dalhousie University, Rm 407, 4th Floor Forrest Building, 5869 University Avenue, PO Box 15000, Halifax, NS B3H4R2 Canada

**Keywords:** Cognitive neuroscience, Motor control

## Abstract

While considered analogous to physical practice, the nature of imagery-based skill acquisition—specifically whether or not both effector independent and dependent encoding occurs through motor imagery—is not well understood. Here, motor imagery-based training was applied prior to or after physical practice-based training to probe the nature of imagery-based skill acquisition. Three groups of participants (*N* = 38) engaged in 10 days of training of a dart throwing task: 5 days of motor imagery prior to physical practice (MIP-PP), motor imagery following physical practice (PP-MIP), or physical practice only (PP-PP). Performance-related outcomes were assessed throughout. Brain activity was measured at three time points using fMRI (pre/mid/post-training; MIP-PP and PP-MIP groups). In contrast with physical practice, motor imagery led to changes in global versus specific aspects of the movement. Following 10 days of training, performance was greater when motor imagery preceded physical practice, although remained inferior to performance resulting from physical practice alone. Greater activation of regions that support effector dependent encoding was observed mid-, but not post-training for the PP-MIP group. Findings indicate that changes driven by motor imagery reflect effector independent encoding, providing new information regarding how motor imagery may be leveraged for skill acquisition.

## Introduction

A glaring difference between motor imagery and physical practice is the lack of sensory feedback related to task performance in motor imagery, as overt movement is absent. Notwithstanding this difference, motor imagery has long been considered analogous to physical practice, including shared neural representations, providing a basis for its effectiveness for driving skill acquisition^1–3^. Contesting this long-standing assumption of functional equivalence however, recent work has suggested that motor imagery results only in the effector independent encoding of a motor program (i.e., referring to global movement features, and the integration of perceptual information to movement goals that are not specific to an effector group^4–9^) as opposed to effector dependent encoding that also occurs in physical practice (i.e., mapping the movement goals and specific movement parameters to the effector to be used in the task)^10–13^. Given that well-established frameworks of skill acquisition indicate both effector independent and dependent encoding are required to acquire general and specific features of a movement in order for proficiency of the motor task to be realized^14–16^, the effector independent nature of motor imagery may explain why motor imagery-based practice leads to less robust improvements in performance when performed in isolation of physical practice^4–6,17,18^.

If motor imagery is effector independent, learning acquired through motor imagery should transfer across effectors after a bout of imagery-based training, and, given that effector dependent encoding leads to improved speed and accuracy with the desired effector, one could envisage motor imagery being more effective when applied prior to physical practice (as opposed to after physical practice) before a certain level of effector dependent encoding has occurred. Indeed, work investigating inter-manual transfer after a bout of motor imagery-based training indicates that performance in both trained and untrained effectors improves^4^, and greater perceptual encoding results relative to physical practice^6^. In contrast, a bout of physical practice-based training leads to inferior performance with the untrained relative to the trained effector^4^ and greater encoding of effector dependent information^6^. In these studies, overall improvements in performance resulting from motor imagery remained inferior to those driven through physical practice^4,6^. In contrast to investigations over short timescales (e.g., single session studies), studies examining parameters of motor imagery-based skill acquisition over periods > 1 week (e.g., multi-session studies) are notably absent. In addition to the paucity of studies examining neural and/or behavioural outcomes associated with motor imagery relative to physical practice over multiple sessions (see^19–21^ for examples), no studies have examined the effect of order when motor imagery is combined with physical practice. Critically, the absence of investigations that evaluate the evolution of both forms of encoding over multiple sessions limits our understanding of how the effector independent nature of motor imagery manifests in both behaviour and brain function, and how motor imagery can be leveraged when applied to learning over periods > 1 week.

Here, participants trained on a complex motor skill (dart throwing), via equivalent amounts of physical practice and motor imagery applied in a different order: the first group performed physical practice followed by motor imagery (PP-MIP), and the second group performed motor imagery followed by physical practice (MIP-PP). We hypothesized that, given an equal dose of motor imagery and physical practice, the greatest gains in task performance should be observed when physical practice-based training is *preceded* by motor imagery-based training. Based on the above theoretical framework, we argue that improvements in performance resulting from physical practice would not be equivalent between groups (i.e., indicating changes overall were due strictly to physical practice), and would instead depend on when motor imagery was applied in the training timeline.

**Figure 1:**
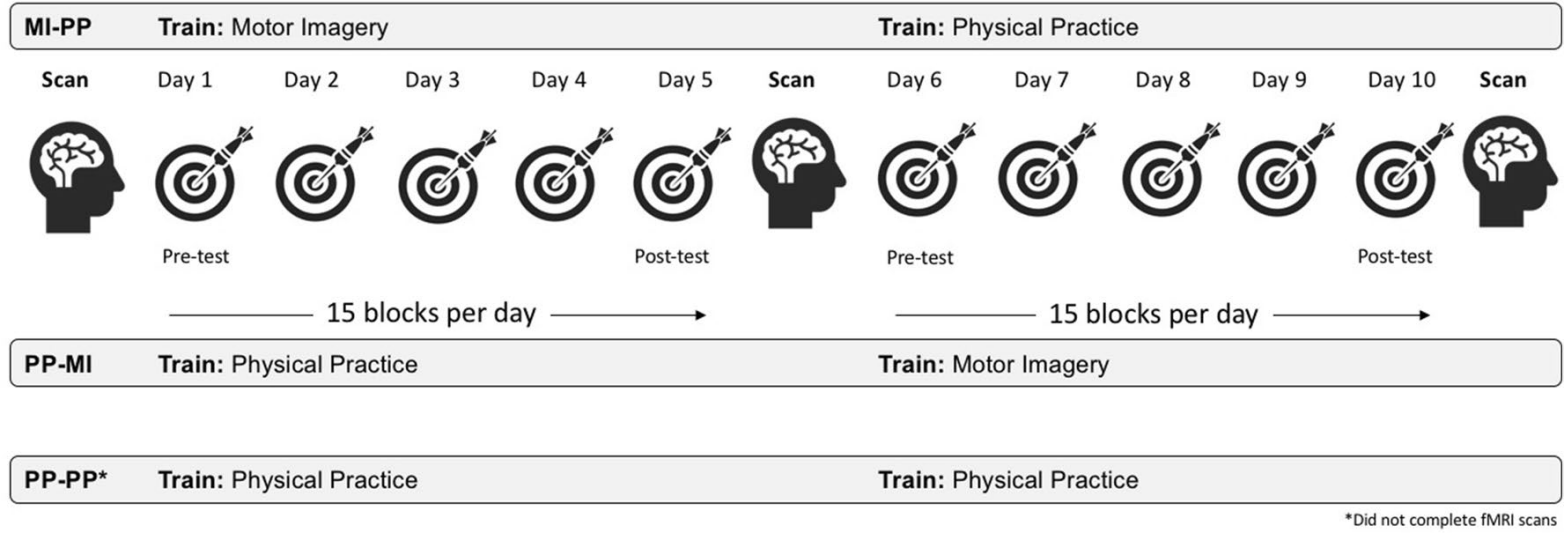
Timeline of the experimental design. Participants engaged in ten training sessions of a dart throwing task, via motor imagery prior to physical practice (MIP-PP), motor imagery following physical practice (PP-MIP), or physical practice only (PP-PP), based on group assignment. Participants completed four physical testing sessions in a pre/post design for each half of the study. Participants in the MIP-PP and PP-MIP groups also underwent three fMRI sessions (pre/mid/post-training) to capture changes in motor imagery-related brain activation of the dart throwing task.

To address our predictions, training-related changes in performance were assessed via physical testing sessions in a pre/post design for each practice modality (i.e., baseline, end of day 5, beginning of day 6, end of day 10; see Materials and Methods for details; Fig. 1). Given the goal of evaluating effector independent and dependent encoding, how each would manifest at the behavioural level was considered. Unlike those typically employed in studies examining learning resulting from motor imagery-based training, here we included a range of assessments to discriminate effector independent from effector dependent encoding. Consequently, encoding of information linked to the global aspects of performance (effector independent)^10,15^ was assessed by measuring spatial consistency and global kinematics which reflect task understanding, intention, and movement goals (i.e., occurring prior to the kinematics-to-dynamics transformation^22,23^), as such features are linked to these internal states versus motor commands^24–26^. Encoding of information linked to muscle-specific commands (effector dependent)^11,15^ was assessed by measuring spatial accuracy and trial-by-trial correction which reflect the movement endpoint and specific movement kinematics. Brain activity during motor imagery of the trained task was also examined, with participants undergoing functional magnetic resonance imaging (fMRI) at three time points (pre/mid/post training). Between- and within-group analyses were performed to characterise functional brain changes during motor imagery of the task as a function of training modality and order over the course of training. A third group of participants did not undergo fMRI but engaged in 10 days of physical practice-based training (PP-PP), which allowed us to anchor our findings to the gold standard of practice.

## Results

### Imagery ability and manipulation checks

Two participants were excluded from the study (one from the MIP-PP group because they dropped out after day 1; and one from the PP-MIP group for excess head motion during the MRI), leaving 36 participants (MIP-PP group: *n* = 11, 7 female; aged 24.5 ± 4.2 years; PP-MIP group: *n* = 11, 8 female; aged 24.6 ± 4.7 years; PP-PP group *n* = 14, 12 female; aged 23.2 ± 3.0 years). Of the remaining participants included in final analyses, one participant from the PP-MIP group engaged in only two of the three blocks during each testing session and had a 4-day break between day 5 and day 6 testing sessions due to technical issues related to the MRI system. One additional participant from the PP-MIP group had an incomplete data set in that the end location of each dart throw was not recorded for day 1, due to experimenter error. No pre-existing group differences in motor imagery ability (MIQ-RS)^27,28^ were observed: there were no significant effects of group (F_1,20_ = 3.34, *p* = 0.08), imagery condition (F_1,20_ = 0.33, *p* = 0.57), or interaction between group and imagery condition (F_1,20_ = 0.33, *p* = 0.57). Further, no differences in motor imagery performance was observed throughout the experiment as determined via manipulation checks of imagery engagement [F_group_(1,20) = 0.44, *p* = 0.52; F_time point_ (2,40) = 0.39, *p* = 0.68; F_interaction_(2,40) = 1.15, *p* = 0.33] and imagery quality [F_group_(1,20) = 2.25, *p* = 0.15; F_time point_ (2,40) = 3.93, *p* = 0.32; F_interaction_(2,40) = 3.93, *p* = 0.47] conducted throughout the neuroimaging sessions. Data related to imagery ability manipulation checks is included in the Supplementary Material (Supplementary Table 1).

### Behaviour

Using standard analysis procedures for dart-throwing tasks, performance was measured in terms of accuracy (radial error; RE) and consistency (bivariate variable error; BVE; see Table 1 for group means)^29–34^. Table 2 shows results from both linear mixed effects models conducted to assess RE and BVE (separate models) as a function of group, time point, and the interaction between group and time point, with participant entered as a random factor. Terms for age and sex were also included in the model (Table 2). Reference levels were set as the PP-PP group (group), and day 1 (time point). Interactions between group and time point were significant (see Table 2) at day 5 for the MIP-PP group indicating that a bout of physical practice led to greater improvements in RE relative to an equivalent bout of motor imagery. Similarly, BVE-related analyses demonstrate a similar trend towards between-group differences at day 5 (observed through an interaction between group and time point for the MIP-PP group) and day 6 (observed through an interaction between group and time point for the PP-MIP group), yet such effects did not achieve statistical significance. Further, while all groups were shown to reach a similar level of skill after training as no group and time point interactions were observed for BVE at day 10, such interaction was trending towards significance for the PP-MIP group. Changes in RE and BVE within each group (i.e., between test sessions) were further characterized via posthoc tests and effect sizes.

**Table 1:** Behavioural data (mean and SD) for task-related performance outcomes (mean radial error; RE, and bivariate variable error; BVE), and autocorrelation lag-1 (ACF1) calculation. Effect sizes characterising changes in performance (day 1 minus day 5; day 6 minus day 10; and day 1 minus day 10) are included. Improvements in performance are noted by negative effect sizes reflecting a decrease in error (mean RE and BVE) and global kinematic variability, and positive effect sizes reflecting an increase in trial-by-trial correction factor (ACF1) and angular velocity. Data for test sessions on day 1 and 5 is previously reported in Kraeutner et al.^40^).

	Test session						
	Day 1	Day 5	d (1 vs. 5)	Day 6	Day 10	d (6 vs. 10)	d (1 vs. 10)
**Mean radial error (cm)**							
MIP-PP	8.70 (3.89)	8.93 (5.08)	0.05	8.37(5.41)	6.23(2.57)	− 0.51	− 0.75
PP-MIP	8.04 (4.05)	5.96 (1.41)	− 0.69	5.75(1.27)	6.68(1.74)	0.61	− 0.44
PP-PP	9.64(2.91)	6.42(1.36)	− 1.42	8.04(1.61)	6.54(1.56)	− 1.18	− 1.33
**Bivariate variable error (cm)**							
MIP-PP	9.69 (4.72)	8.58 (3.84)	− 0.26	8.30(3.33)	6.55(2.82)	− 0.57	− 0.81
PP-MIP	9.19 (4.92)	5.72 (1.24)	− 0.97	5.97(1.15)	6.91(2.35)	0.51	− 0.59
PP-PP	10.08 (3.98)	6.55(1.40)	− 1.18	9.22 (2.94)	6.77 (1.61)	− 1.04	− 1.09
**ACF1**							
MIP-PP	0.19 (0.07)	0.14 (0.10)	− 0.56	0.10 (0.08)	0.14 (0.10)	0.41	− 0.56
PP-MIP	0.15 (0.13)	0.14 (0.12)	− 0.07	0.14 (0.11)	0.18 (0.10)	0.39	0.28
PP-PP	0.16 (0.12)	0.19 (0.17)	0.16	0.22 (0.17)	0.24 (0.15)	0.14	0.58
**Global kinematic variability**							
MIP-PP	4.43 (2.1)	3.32 (1.3)	− 0.52	4.43 (3.0)	3.19 (1.8)	− 0.58	− 0.58
PP-MIP	5.21 (2.3)	2.77 (2.0)	− 1.36	3.23 (1.5)	2.78 (1.2)	− 0.26	− 1.36
PP-PP	4.78 (1.5)	3.53 (2.1)	− 0.59	5.08 (2.9)	3.57 (1.8)	− 0.71	− 0.57
**Angular velocity**							
MIP-PP	334.5 (95.2)	365.6 (115.4)	0.30	370.7 (111.1)	364.2 (93.7)	− 0.07	0.32
PP-MIP	289.1 (95.7)	350.9 (121.9)	0.55	359.8 (103.8)	360.3 (123.7)	0.01	0.63
PP-PP	328.9 (61.9)	347.5 (113.6)	0.21	334.2 (85.3)	348.9 (81.8)	0.18	0.28

**Table 2:** Linear mixed effects conducted to assess changes related to radial error and bivariate variable error.

Predictors	**Radial error (cm)**			**Bivariate variable error (cm)**		
	Estimates	CI	*p*	Estimates	CI	*p*
(Intercept)	12.87	8.52–17.23	** < 0.001**	11.71	7.23–16.19	** < 0.001**
MIP-PP group	− 0.43	− 2.38–1.52	0.667	0.05	− 2.31–2.42	0.964
PP-MIP group	− 0.80	− 2.78–1.18	0.428	− 0.21	− 2.60–2.18	0.862
Day 5	− 3.19	− 4.28 to −2.10	** < 0.001**	− 3.47	− 5.27 to −1.68	** < 0.001**
Day 6	− 1.58	− 2.67 to −0.49	**0.004**	− 0.81	− 2.60–0.99	0.378
Day 10	− 3.07	− 4.16 to −1.98	** < 0.001**	− 3.26	− 5.05 to −1.47	** < 0.001**
Male	− 2.26	− 3.86 to −0.66	**0.006**	− 2.47	− 4.09 to −0.84	**0.003**
Age	− 0.13	− 0.31–0.05	0.168	− 0.06	− 0.24–0.12	0.534
MIP-PP group * Day 5	3.41	1.79–5.04	** < 0.001**	2.36	− 0.30–5.03	0.083
PP-MIP group * Day 5	1.10	− 0.55–2.74	0.191	0.00	− 2.66–2.67	0.999
MIP-PP group * Day 6	1.23	− 0.39–2.86	0.137	− 0.58	− 3.25–2.08	0.668
PP-MIP group * Day 6	− 0.83	− 2.47–0.81	0.322	− 2.41	− 5.08–0.25	0.076
MIP-PP group * Day 10	0.59	− 1.04–2.21	0.478	0.12	− 2.55–2.79	0.930
PP-MIP group * Day 10	1.63	− 0.01–3.28	0.051	0.98	− 1.69–3.64	0.473
**Random effects**						
σ^2^	30.48			5.58		
τ_00participantNum_	3.75			2.96		
ICC	0.11			0.35		
N_participantNum_	36			36		
Observations	2091			143		
Marginal R^2^ /Conditional R^2^	0.076/0.177			0.283/0.532		

Regarding the PP-PP group (our gold standard control group), significant changes (p < 0.05) observed from our posthoc tests, supported with large effect sizes, were observed between each time point demonstrating robust improvements in both halves of training (day 5 vs 1; day 10 vs 6), as well as overall (day 10 vs 1; Table 1). With respect to improvements in performance observed in our groups that engaged in both modalities of practice, the PP-MIP group showed significant improvements for both RE and BVE only in the first half of training (i.e., during physical practice) but not the second half (during motor imagery). Such improvements are further supported by a moderate (RE) and large (BVE) effect size between day 5 vs 1. In contrast however, moderate positive effect sizes for both RE and BVE were obtained between day 10 vs 6 indicating a worsening in performance in the second half of training (i.e., through motor imagery) for this group. In contrast, the MIP-PP group showed significant improvements only in the second half of training, and only for RE. Yet, a small effect size obtained between day 5 vs 1 indicates improvement in the first half of training for BVE, and moderate effect sizes obtained between day 10 vs 6 for both RE and BVE indicate improvement in the second half of training. Interestingly however, while the MIP-PP and PP-MIP groups showed no significant differences for RE and BVE between days 5 and 6, significant differences were observed in the PP-PP group (*p* = 0.019), indicating a worsening in performance over the retention period.

**Figure 2:**
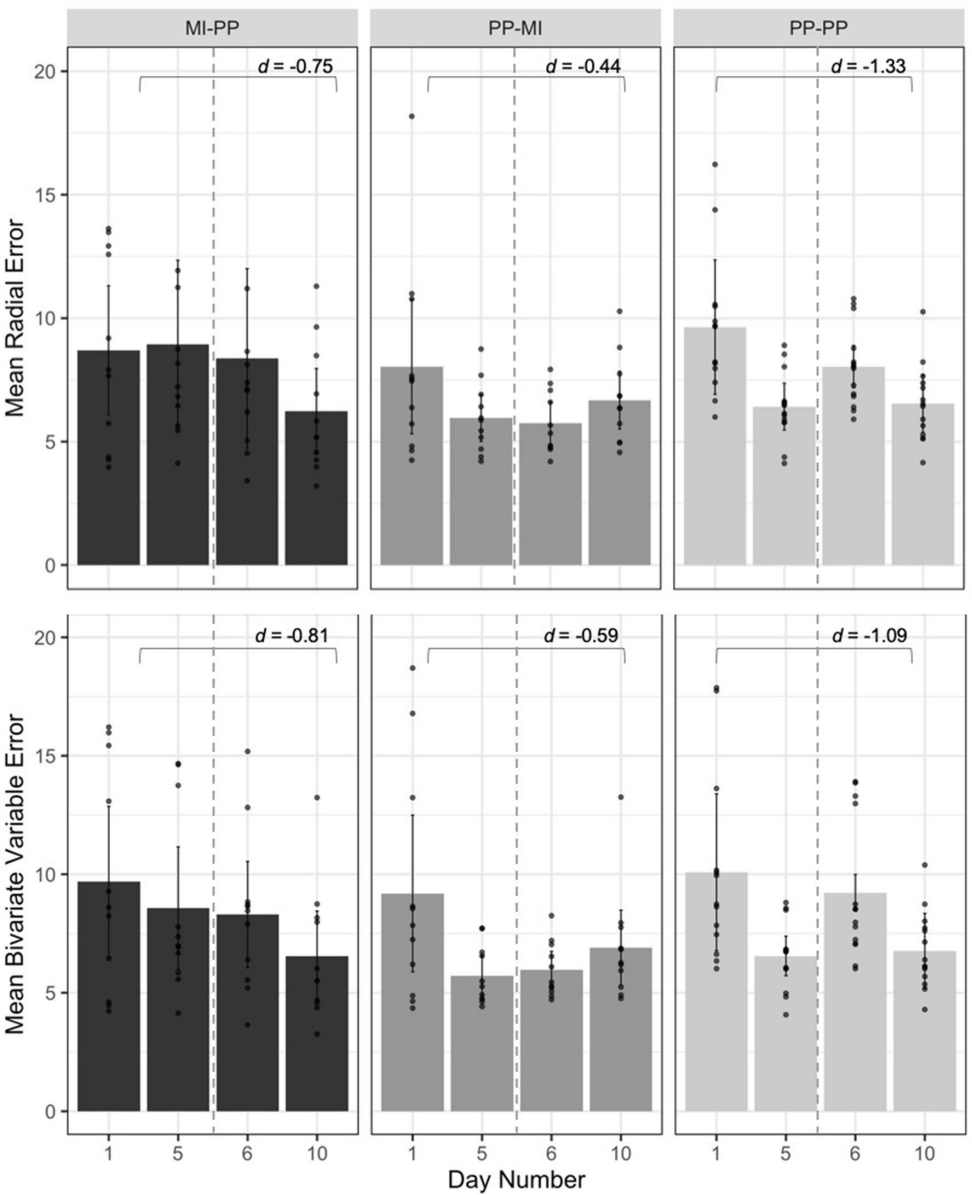
Change in task-related performance outcomes. Mean radial (top) and bivariate variable (bottom) error are shown across groups for each testing session. Individual participant means are overlaid, with error bars denoting 95% confidence intervals (see Table 1 for mean values and standard deviations). Effect sizes characterising overall improvements in performance (day 10 minus day 1; Cohen’s d) are shown for each group. While inferior to improvements achieved by physical practice alone (PP-PP), greater improvements in performance were observed when motor imagery was applied prior to physical practice (MIP-PP) compared to after physical practice (PP-MIP).

With respect to differences observed overall (day 10 vs day 1), the MIP-PP group showed robust overall improvements in RE and BVE (p_RE_ = 0.003, d_RE_ =  − 0.75; p_BVE_ = 0.013, d_BVE_ =  − 0.81), yet the PP-MIP group did not (trending, p_RE_ = 0.050, d_RE_ =  − 0.44; p_BVE_ = 0.122, d_BVE_ =  − 0.59). Relative to results from the PP-PP group, overall improvement in performance achieved by the MIP-PP group was still less than that achieved by the gold standard (p_RE_ < 0.001 d_RE_ =  − 1.33; p_BVE_ < 0.001, d_BVE_ =  − 1.09). All resultant values from posthoc analyses conducted related to RE and BVE are included as Supplementary Materials (Supplementary Table 2). See Fig. 2. for a visual depiction of changes in task-related performance outcomes.

#### ACF1

To add resolution to the analysis of performance, we applied a lag-1 autocorrelation (ACF1) calculation to determine the correction factor between throws on a trial-by-trial basis (using the mean X and Y deviation from the origin following separate calculations in the X and Y plane, described in Materials and Methods; see Supplementary Fig. 1 for a visualization)^35,36^. A high correlation at lag-1 implies the use of throw-by-throw feedback—an ability gained as a forward model process is established through effector dependent encoding^34^. Critically, such analysis permits the investigation of an aspect of performance that is not discernible from measures collapsed across time. Interestingly, while a main effect of group on ACF1 was observed (F_2,131_ = 3.21, *p* = 0.04), no effect of time point, nor interaction between group and time point was observed (F_3,131_ = 0.56, *p* = 0.65; F_6,131_ = 0.82, *p* = 0.56, respectively). No significant effects were observed in our posthoc contrasts (p > 0.05) conducted to further examine the main effect of group, although a group difference between the MIP-PP and PP-PP group was trending (p = 0.058; Supplementary Table 3). Effect sizes calculated to characterise ACF1 changes driven via training separately for each group (see Table 1) however revealed that changes in ACF1 were only observed in the second half of training regardless of order of modality (as evidenced via moderate effect sizes in the second half of training—day 6 vs. day 10—in the MIP-PP and PP-MIP groups). Overall (day 1 vs. day 10), the PP-PP group demonstrated the greatest improvement in their ability to correct between throws on a trial-by-trial basis (evidenced via a large effect size; Table 1).

#### Kinematic variability

Training-related changes in kinematics were assessed via analysis of 2D images captured during the test sessions. In particular, global kinematic variability (comprised of variability of shoulder angle at both the ‘take back’ and the point of release for each dart throw as well as elbow angle at release; see Methods) and angular velocity (defined as the change in elbow angle over throwing time) were obtained at each time point (Table 1). Only a significant main effect of time point was observed [F(3,96) = 11.31, *p* < 0.001], indicating that global kinematic variability decreased with training. No main effect of group nor interaction between group and time point was observed [F(2,32) = 0.65, *p* = 0.53; F(3,96) = 1.54, *p* = 0.20; corrected using Greenhouse–Geisser estimates of sphericity]. We observed a significant decrease in global kinematic variability between day 5 versus 1 (i.e., the first half of training) and between day 10 versus 1 (i.e., overall) via posthoc contrasts conducted to further investigate the main effect of time point (Supplementary Table 3). Effect sizes calculated to characterise the change in kinematic outcomes across sessions show that global kinematic variability decreased via motor imagery, regardless of when it was applied in training (Table 1). The changes driven via motor imagery applied at any point in training remained inferior to changes driven by strictly physical practice-based training, as evidenced by a large effect size overall (day 1 vs. day 10) for the PP-PP group relative to moderate effect sizes observed in the PP-MIP and MIP-PP groups (Table 1). A similar trend was observed for angular velocity: we observed a significant main effect of time point [F(3,96) = 5.56, *p* = 0.003, yet no main effect of group [F(2,32) = 0.15, *p* = 0.86] nor significant interaction between group and time point [F(6,96) = 1.23, *p* = 0.30, corrected using Greenhouse–Geisser estimates of sphericity]. Thus, angular velocity increased after training regardless of modality. While no significant effects were observed in our posthoc contrasts (p > 0.05) conducted to further examine the main effect of time point (Supplementary Table 3), effect sizes showed that angular velocity increased primarily in the early stages of training (i.e., days 1–5; Table 1).

#### fMRI

**Figure 3:**
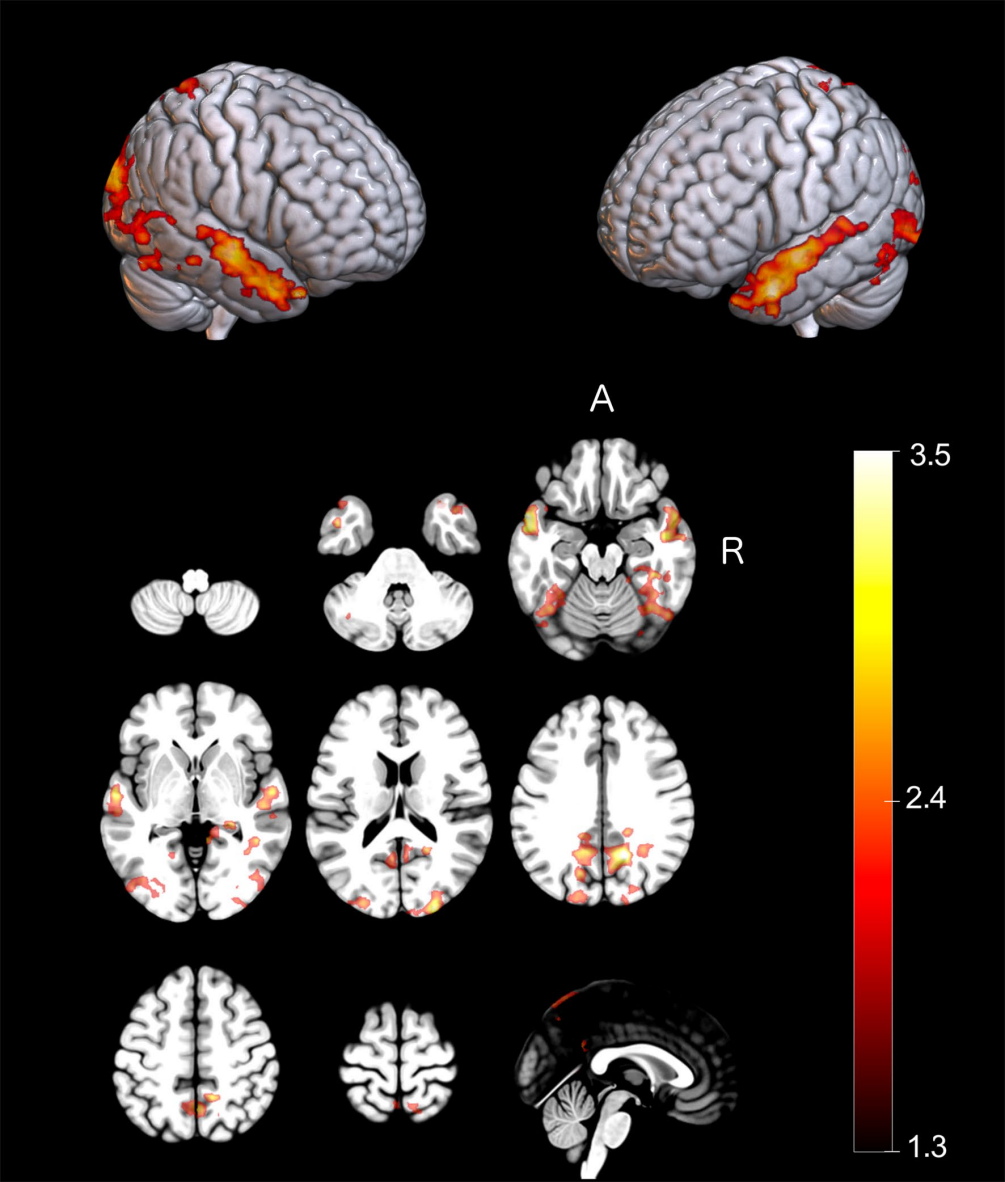
Effects of training on resultant motor imagery-based brain activity where the colourbar represents the Z-max value. Activated voxel clusters remaining after subtraction of the map of activations of post-training scan from the pre-training scan across all participants. Activation was localized to regions including bilateral fusiform gyri, precuneus, middle temporal gyri, and middle occipital gyri. Clusters shown reached a cluster corrected threshold (Z > 2.0; *p* < 0.05), family-wise error corrected for number of comparisons, and are shown overlaid on the MNI template.

Following standard preprocessing procedures^37–39^, between and within-group comparisons were conducted using statistical activation maps across runs for each individual and time point to characterise functional brain changes during motor imagery of the dart throwing task. Within-group comparisons (pre vs. mid; mid vs. post) indicated that training-related changes in brain activation that achieved statistical significance were only driven via physical practice, and only during the initial stage of training. This analysis is included as Supplementary Material (see Supplementary Table 4)—for an in-depth discussion of the impact of training-modality on resultant patterns of motor imagery-based brain activity, see^40^. As all groups demonstrated similar improvements in performance over the course of the study, we conducted a comparison of brain activation from pre- to post-training (i.e., post- > pre-training scan) across all subjects, revealing greater activation localized to regions including bilateral precuneus, fusiform gyri, middle temporal gyri, and occipital regions at the post-training scan (post > pre; Fig. 3; Table 3).

**Table 3:** MNI coordinates of the local maxima of regions activated during motor imagery of the dart throwing task across all participants (only coordinates from peak voxel are shown). Comparisons were conducted to assess differences in motor imagery-related brain activation following training.

Anatomical region	MNI coordinates (mm)			Z-score
	x	y	z	
**Post > pre-training**				
L Precuneus	− 12	− 57	41	3.46
L Fusiform gyrus	− 31	− 62	− 13	3.38
L Inferior occipital gyrus	− 29	− 75	− 5	2.86
L Middle temporal gyrus	− 52	8	− 26	3.93
L Superior temporal gyrus	− 54	− 5	− 8	3.68
R Cuneus	18	− 57	36	3.97
R Precuneus	19	− 50	41	3.46
R Superior occipital gyrus	27	− 96	17	3.36
R Fusiform gyrus	36	− 61	− 11	3.07
R Middle temporal gyrus	54	− 11	− 11	3.77
R Superior temporal gyrus	56	− 10	− 5	3.73
R Middle temporal pole	32	18	− 34	3.6
R Superior temporal pole	38	10	− 27	3.53

No significant differences in activation were observed for pre > post-training.

**Figure 4:**
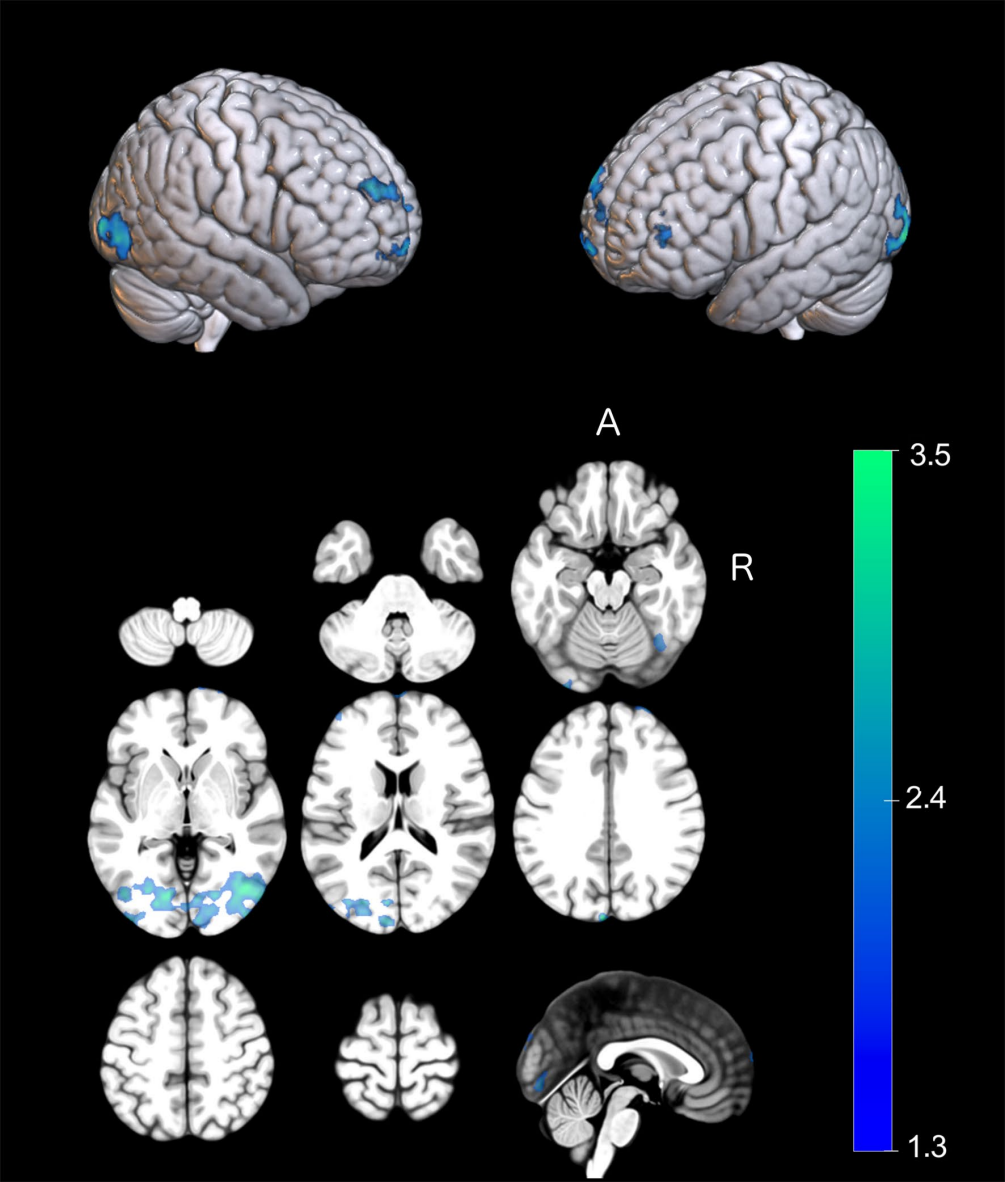
Correlation between brain activity and performance (bivariate variable error) at the post-training scan, where the colour bar represents the Z-max value. During motor imagery, bivariate variable error correlates negatively with brain activity localized to occipital regions as well as left superior and bilateral middle frontal gyri. Clusters shown reached a cluster corrected threshold (Z > 2.0; *p* < 0.05), family-wise error corrected for number of comparisons, and are shown overlaid on the MNI template.

Between-group comparisons, adjusted for BVE, were conducted at each scan to assess the impact of training modality on resultant patterns of motor imagery-related brain activity. At the pre-training scan, additional activation was observed for the MIP-PP group, localized to the ipsilateral cerebellum (MIP-PP > PP-MIP; Table 4). At the mid-training scan, additional activation was observed for the PP-MIP group (PP-MIP > MIP-PP), localized to regions including the supplementary motor area (SMA), anterior cingulate cortex, cerebellum, contralateral precentral and middle temporal gyri, and ipsilateral superior frontal gyrus. Negative correlations between BVE and motor imagery-related activity were found in regions including bilateral parietal cortices and lingual gyri (Fig. 4; Table 4). At the post-training scan, no differences that reached statistical significance were observed, but negative correlations between BVE and motor imagery-related activity were found in regions including bilateral frontal and occipital regions (Table 4).

**Table 4:** MNI coordinates of local maxima resulting from between-group comparisons conducted at each time point (only coordinates from peak voxel are shown).

Anatomical region	MNI coordinates (mm)			Z-score
	x	y	z	
*Pre-training*				
**MIP-PP > PP-MIP**				
R Cerebellum (lobule VI)	24	− 59	− 34	3.25
R Cerebellum (lobule VIIb)	26	− 77	− 47	3.38
R Cerebellum (lobule VIII)	22	− 59	− 37	3.13
R Cerebellum (crus I)	39	− 88	− 32	2.85
R Cerebellum (crus II)	28	− 90	− 40	3.12
**PP-MIP > MIP-PP**				
–	–	–	–	–
*Mid-training*				
**MIP-PP > PP-MIP**				
–	–	–	–	–
**PP-MIP > MIP-PP**				
L Superior temporal pole	− 36	27	− 26	3.73
L Middle temporal gyrus	− 67	− 10	− 2	3.53
L Cerebellum (lobule VI)	− 26	− 59	− 29	3.22
L Cerebellum (lobule IV/V)	− 4	− 59	− 18	3.06
L Anterior cingulate	0	8	26	3.39
L Medial frontal gyrus	− 11	34	35	2.81
L Precentral gyrus	− 30	− 16	76	3.23
L SMA	− 11	− 5	73	3.22
L Paracentral lobule	− 16	− 20	77	3.13
R Cerebellum (lobule IV/V)	10	− 46	− 7	3.2
R Cerebellum (vermis IV/V)	4	− 58	− 16	3.1
R Anterior cingulate	10	12	30	2.81
R SMA	2	− 3	58	2.73
R Superior frontal gyrus	16	− 3	74	3.11
**Positive BVE effect**				
–	–	–	–	–
**Negative BVE effect**				
L Superior parietal lobule	− 18	− 51	51	3.52
L Precuneus	− 18	− 51	51	2.91
L Lingual gyrus	− 8	− 59	53	3.34
L Calcarine	− 20	− 74	0	3.07
R Superior parietal lobule	21	− 66	64	3.45
R Angular gyrus	40	− 60	30	3.32
R Lingual Gyrus	19	− 77	− 1	3.05
*Post-training*				
**MIP-PP > / < PP-MIP**				
–	–	–	–	–
**Positive BVE effect**				
–	–	–	–	–
**Negative BVE effect**				
L Middle occipital gyrus	− 46	− 96	− 3	4.42
L Cuneus	− 5	− 93	32	3.70
L Middle frontal gyrus	− 34	65	24	3.79
L Superior frontal gyrus	− 31	65	26	3.55
R Inferior occipital gyrus	41	− 75	− 4	3.69
R Middle frontal gyrus	30	68	26	4.22

Instances in which no significant differences in activation were noted are indicated by dashed lines.

## Discussion

In manipulating the order of motor imagery and physical practice in training, our findings add to a growing body of evidence suggesting that motor imagery is not functionally equivalent to physical practice which has been the consensus view for some time^1,2,41,42^. Below we discuss our findings, that motor imagery facilitates effector independent encoding, reflected in global versus specific aspects of movements, and that the effector independent nature of motor imagery may thus lend itself to being most effective when applied prior to physical practice. Finally, we broadly discuss important considerations for the motor learning field, stemming from nuances observed in our findings.

### Effector independent versus effector dependent encoding

Our argument, that motor imagery operates via effector independent encoding, arises from the finding that motor imagery led to improvements in consistency, and larger overall improvements in performance were observed (i.e., after all ten practice sessions) when motor imagery was applied prior to physical practice rather than its alternative. Further, motor imagery led to decreases in global kinematic variability, regardless of where it was applied in practice. These global improvements reflect the ongoing processing of environmental and sensory stimuli of the movement and integration with an understanding of movement elements, and task goals to create a motor representation^14,16^. Further, we did not observe further improvements in consistency or accuracy when motor imagery was applied following physical practice. Several studies support the effector independent nature of motor imagery, showing that although motor imagery creates elaborate mental representations of a movement (i.e., a number of different movement ‘chunks’ termed basic action concepts that are organized into a hierarchical structure as expertise is gained)^17,43,44^, such enhancement of the mental representation does not necessarily manifest at the behavioural level^41^. Indeed, robust gains driven by motor imagery are noted across literature employing key-press sequence tasks where effector independent encoding is critical^45^, whereas marginal or no improvements are noted in performance of discrete key-presses (i.e., overall reaction times vs. sequence-specific, reflecting effector-specific encoding)^46^. Further, fronto-parietal regions implicated in effector independent encoding^10,12,47^ are shown to be consistently activated during motor imagery^48,49^. Thus, as in the current study, when motor imagery is applied after physical practice it is likely that the aforementioned effector independent processing and associated refinements to the motor program do not translate to further improvements in task-related outcomes.

With respect to effector dependent encoding, we did not observe changes in specific aspects (i.e., RE or ACF1) of performance driven via motor imagery applied at any point in the study, adding to a growing body of evidence suggesting that motor imagery is not functionally equivalent to physical practice. Changes related to RE and the ACF1 reflect the onset of effector dependent encoding, whereby the changes observed from trial-to-trial represent the well-established error detection/correction mechanism that facilitates the ideal kinematics of the end-effector and allow for the task goal to be achieved (i.e., the final position of the dart)^50^. Further, greater activation localized to the SMA, cerebellum, and anterior cingulate cortex observed following an equivalent bout of motor imagery- versus physical practice-based training (i.e., at the mid-training scan^40^) supports the notion that physical practice is required to facilitate effector dependent encoding. Specifically, these regions are implicated in both the ‘kinematics-to-dynamics’ transformation (i.e., reflecting the dynamics of the upcoming movement and commands sent to the effectors)^22,51,52^, and error detection/correction mechanisms that rely on sensory reafference to disengage in the incorrect response^53,54^ and update the motor program^55–59^. Our findings are also consistent with literature showing inhibition of primary motor cortex, a brain region that supports effector dependent encoding evidenced from investigations of physical practice^10,12^, does not impact motor imagery-based learning^5^ and activation localized to this region is inconsistently reported in the motor imagery neuroimaging literature^48,60,61^. Thus, unlike physical practice, the motor program is not mapped to a specific effector through motor imagery.

How motor imagery facilitates the development of the motor program, as the absence of overt movement precludes the use of sensory feedback in an error detection/correction mechanism^45,62–64^, remains a key question—a query in line with a theoretical framework proposing that motor imagery arises through central executive functions that are not required during physical performance (the ‘motor-cognitive model’)^65^. Interestingly, a system comparing visual feedback to simulated visual consequences is critical to planning motor actions (during physical practice) prior to effector selection (i.e., mapping motor representations to an effector), separate from that which generates the efference copy and intended consequences of the movement^66^. In showing that activation in regions previously thought to reflect the reliance on a visual image during motor imagery of a task^67^ negatively correlated with improvements in performance (i.e., bilateral parietal cortices and lingual gyri at the mid-training scan; and occipital regions as well as left superior and bilateral middle frontal gyri at the post-training)^40^, our findings may suggest that previously theorized error detection/correction mechanisms active during motor imagery^18,68^ only supports refinement of effector independent representations, relying on these visual comparisons. This argument is further substantiated by our finding of increased activation localized to occipital–temporal areas observed following training regardless of the order that motor imagery was applied, in line with work examining activation during (imagery-based) pre-shot routines in expert versus novice archers^69^ and golfers^70^. Activation of these regions, associated with task expertise, is thought to reflect greater visual control and perceptual learning^69–71^, further pointing to a reliance on regions critical to central executive functions as suggested in the motor-cognitive model^65^.

### Motor imagery as a scaffold

Alternatively, it may well be that motor imagery was ineffective throughout practice and the observed improvements in performance were driven by physical practice alone. In this scenario, the poorer outcomes observed in the PP-MIP group (relative to MIP-PP at day 10; Fig. 2) would be attributable to the deterioration of skill retention during the ‘rest’ (i.e., motor imagery) interval in the second half of the study. When examined more closely, that there appeared to be no added effect of motor imagery when it was applied prior to physical practice is surprising; indeed, relative to 5 days of physical practice-based training (i.e., day 5 vs. 1 in the PP-MIP and PP-PP groups), MIP-PP overall (i.e., day 10 vs. 1 in the MIP-PP group) did not produce greater improvement in performance as assessed via effect sizes, or via additional statistical testing conducted to probe between group differences in the relevant change scores (included in Supplementary Materials; see Supplementary Figs. 2 and 3, and Supplementary Tables 6 and 7). However, nuances in our findings suggest that while physical practice is the gold standard for skill acquisition when employed alone, mixed modality approaches (i.e., MIP + PP) employed in training are not equal. Specifically, a direct comparison of effects driven by physical practice indicates that gains in performance resulting from physical practice in the second half of training (i.e., days 6 vs. 10 in the MI-PP group) were less than gains resulting from physical practice in the first half of training (i.e., day 1 vs. 5 in the PP-MI and PP-PP groups). Yet, overall gains in performance were greater in the MIP-PP relative to the PP-MIP group, and similar to gains observed in the PP-PP group, leading to the conclusion that gains in performance observed in the MIP-PP group must have occurred due to some benefit associated with the application of motor imagery in the first half of training.

While the findings of the current work do not definitively support an added benefit of motor imagery practice preceding physical practice, they do represent an important step related to how motor imagery may be leveraged in longer-term practice; the nuances in performance outcomes observed between groups and time points generated broader questions about motor imagery including what outcome measures are best to capture motor imagery-based training effects, and optimal practice schedules (and dose) of motor imagery to be deployed. Namely, that motor imagery relies on effector independent encoding warrants an evaluation of outcome measures used in the literature—outcomes typically used to measure physical practice (i.e., effector dependent encoding) driven improvements may not capture the true effects driven by motor imagery. Thus, a range of outcome measures encompassing such global aspects (i.e., those more related to spatial consistency) must be applied to capture motor imagery-based training effects. Related to practice schedules, here we employed an equivalent dose of motor imagery and physical practice, administered in five consecutive sessions, with results showing larger overall improvements in performance were observed (i.e., after all ten practice sessions) when motor imagery was applied prior to, rather than following, physical practice. Given the dearth of knowledge related to ideal parameters of motor imagery-based training (see^72–74^ for examples of work regarding imagery type and session duration), more research exploring the effect of motor imagery when combined with physical practice over different timescales and order is required to elucidate an optimal motor imagery and physical practice combination. To suggest that motor imagery cannot be leveraged to optimize physical practice-based training in light of a single study would be shortsighted. Indeed, examining the effect of combinations of motor imagery and physical practice is important as the notion that motor imagery is largely ineffective once physical practice has occurred is counterintuitive given the effective use of motor imagery as a practice modality in multiple disciplines (see^3,45^ for examples). It may well be that suggested scaffolding effects of motor imagery are enhanced when motor imagery is layered with physical practice—for instance, if motor imagery preceded physical practice within each day of training, perhaps the more elaborate effector independent representations^17,43^ resulting from motor imagery would be leveraged more effectively for effector dependent encoding to occur via physical practice—leading to greater gains in performance than that occurring through physical practice alone.

Interestingly, expertise is thought to modulate gains in performance driven by motor imagery (see motor simulation and performance model)^75^ whereby physically skilled individuals (experts) performance benefits from motor imagery to a greater extent than that of a novice^75^. The greater benefit of motor imagery enjoyed by experts relative to novices is that a motor program consolidated in memory (through repeated physical execution) may be more readily accessible by experts^75^, which in turn permits motor imagery to act upon the motor system by enhancing the motor program at the perceptual level, ultimately driving improved performance^17,43^. Indeed, experts have more focal activity localized to contralateral sensorimotor regions during motor imagery of their expert task that may reflect their ability to facilitate effector dependent encoding through this practice modality^76,77^. We contend that without such information readily available or robust motor programs consolidated in memory, novices can only update effector independent representations during motor imagery. Thus, any improvements in performance are linked to regions underlying visual and/or perceptual processes as noted above. Further, unlike the range of assessments employed here, those typically included in motor imagery studies are arguably suited to capture changes in performance that manifest from effector dependent encoding, and thus fail to capture performance gains driven by motor imagery in novices. This discord may account for inconsistency in performance gains resulting from motor imagery-based training, particularly amongst novices (see^45,75^ for respective reviews)—an important implication for the way in which motor imagery-based skill acquisition is probed in future studies. Future work examining scaffolding effects of motor imagery in over longer timescales of practice and employing a range of assessments is critical to understanding the extent to which motor imagery facilitates effector independent versus dependent encoding.

### Considerations

Why we did not observe changes in motor imagery-related brain activity driven in the second half of training is likely attributable to methodological limitations: namely, that BOLD indirectly reflects an increased use of neural substrates and is limited by its low temporal resolution^74^. Studies employing electrophysiological measures report both a smaller magnitude and shorter period of activation during motor imagery relative to physical practice of the same task^79–81^. As we employed a block design (30 s blocks, mirroring the blocks during training and testing) in the fMRI experiment, it is possible that any lasting changes in motor imagery-related brain activation driven by training may be too minimal or not sustained long enough across individuals to be observed via fMRI, and particularly at a group-level^79,82^. Alternatively, changes in brain activity during the slow stages of learning, required to achieve skill proficiency in complex skills (i.e., to reach the autonomous stage of skill acquisition^16^) may require months or years of practice^83^. As none of our participants achieved an expert-level of proficiency on the task, the lack of changes observed in motor imagery-related activation may also be attributed to a greater dose and timescale required to drive such changes.

While the MIP-PP and PP-MIP groups showed no differences in performance outcomes between days 5 and 6 (i.e., indicating a retention effect), a difference was observed in the PP-PP group, indicating a worsening in performance following this retention interval (i.e., mid-point break, to allow for neuroimaging to occur in the mixed modality groups). As the PP-PP group did not engage in any motor imagery (i.e., during the fMRI sessions), the retention effects observed in the MIP-PP and PP-MIP groups may be related to enhanced consolidation induced by the additional motor imagery training in these groups^45,84^. Further, while the addition of motor imagery in these groups was albeit a relatively small dose, work has shown that as little as 25 trials can lead to improvements in performance^85^, thus enhancing the stability of improvements in performance observed in the mixed modality groups.

In summary, relative to physical practice, whereby both effector independent and dependent encoding occurs, our findings add to a growing body of evidence suggesting that motor imagery is not functionally equivalent to physical practice. Here, we show that motor imagery facilitates encoding of effector independent representations, reflected in improvements in global versus specific aspects of movements. Further, our results indicate that mixed-modality approaches in training are not all equal: motor imagery applied prior to physical practice led to greater improvements in performance than its alternative. However, nuances in these data, in particular improvements driven across each time point of training, suggest a need to explore broader questions about how the effects of motor imagery are captured, as well as practice schedules and dose in which combinations of motor imagery and physical practice result in maximized motor learning.

## Materials and methods

### Participants

Thirty-eight participants (right handed, as determined by a score of ≥ 40 on the Edinburgh Handedness Inventory)^86^ were recruited from the local and university community. The Nova Scotia Health Authority research ethics board approved the study, all participants gave written consent after being informed of the experimental procedures, and the experiment was conducted in accordance with the principles of the Declaration of Helsinki. All participants were healthy, reported normal hearing, were free of neurological disorders, and had no contraindications to MRI. All participants underwent 10 total practice sessions of a dart-throwing task, according to group assignment: motor imagery prior to physical practice (“MIP-PP”, motor imagery following physical practice (“PP-MIP”), or physical practice for all sessions (“PP-PP”). The data reported herein is an extension of work reported in^40^, whereby participants engaged in 5 days of motor imagery or physical practice-based training. Thus, the participants included in this study in the MIP-PP and PP-MIP groups are from the “MI” and “PP” groups in the study reported in^40^, respectively.

### Experimental design

Training and testing sessions followed procedures as described in^40^ (Fig. 1). Briefly, each training session lasted ~ 20 min and involved 15 blocks of dart throws, with six trials (dart throws) per block. Dart throwing was performed in accordance with World Dart Federation regulations^87^, and participants were instructed to aim at the bullseye and to limit their throws to flexion and extension movements at the elbow in the sagittal plane. At the outset of training, all participants underwent a brief familiarization phase, involving a 5-min video that provided exemplar performances, both male and female from both the third- and first-person perspective. Participants performed dart throws using nickel/brass tipped darts that weighed 22 g (physical practice sessions), or imagined performing throws cued by an auditory script delivered via noise-cancelling headphones (motor imagery sessions). At the outset of each motor imagery session, participants were first oriented to and instructed to perform kinaesthetic motor imagery (i.e., 1st person perspective with an emphasis on the polysensory aspect of the task)^74,88^. Physical test blocks comprised 18 total dart throws, performed in three blocks of six throws, lasting ~ 15 min, embedded at four points throughout training on days 1, 5, 6, and 10; Fig. 1 (similar to parameters employed in^30,33,89^. The final location of each dart was digitized (Polhemus Fastrak, Colchester, VT) and video data were recorded to capture participant kinematics in the sagittal plane (Canon Powershot SX280 HS mounted perpendicular to the throwing line; Canon Canada, Inc.), and stored for offline analysis. Training sessions within either half of the study (days 1–5 or days 6–10) were scheduled within 10 days (i.e., averaging no more than 2 days apart; with the exception of one participant in the MIP-PP group who had one training session that occurred 4 days from the prior due to a scheduling conflict) and the ‘break’ between testing sessions on day 5 and day 6, for which participants in the MIP-PP and PP-MIP groups underwent an fMRI session.

### MRI acquisition

Structural and functional MRI data were acquired on a 3 T GE MRI (GE Medical Systems, Waukesha, WI, with a 32 channel RF Head Coil). A 3D T1-weighted anatomical image was acquired using a IR-prepped fast spoiled gradient recalled echo (IR-FSPGR) sequence (inversion time (TI) = 450 ms, repetition time (TR) = 4.0 ms, echo time (TE) = 1.33 ms, flip angle = 9°, field of view (FOV) 25.6 cm, 256 × 256, 184 sagittal slices at 1 mm thickness, auto-calibrating reconstruction for cartesian imaging (ARC) phase acceleration = 1, ARC slice acceleration = 1). A T2-weighted anatomical image was acquired using a 3D CUBE sequence (inversion time (TI) = 400 ms, TR = 4200 ms, TE = 101 ms, 140 Echo Train Length, 25.6 cm FOV, 256 × 256, 184 sagittal slices at 1 mm thickness, ARC phase acceleration = 1.5, ARC slice acceleration = 1). Functional MRI data were acquired using a 2D multi-band echo-planar image (EPI) sequence (TR = 950 ms, TE = 30 ms, flip angle = 60°, 21.6 cm FOV, 72 × 72, 3 mm thick slices, 224 volumes, MUX acceleration factor 3 slice direction, ARC acceleration factor 2 in-plane; Stanford Center for Cognitive and Neurobiological Imaging, http://cni.stanford.edu). Additional EPI reference scans with matching parameters except phase-encode blip direction reversal were acquired to facilitate field distortion correction^39,90^.

The fMRI experiment included four runs, each consisting of two motor imagery blocks (28.5 s) with alternating rest blocks (19 s; eyes open). Each run began and ended with a rest block. The start of each motor imagery block was cued visually, and an auditory cue signified the end of each block. Participants were asked to visualize throwing six darts (equivalent to one training block) using kinaesthetic imagery with their eyes closed. Participants were cued to the completion of the imagery block via an auditory tone. Manipulation checks were administered after each run inquiring about the participants’ level of engagement and the quality of imagery on a scale of 1 (not engaged; poor quality) to 5 (extremely engaged; excellent quality). Stimuli were presented using Presentation software (Neurobehavioral Systems, Inc. Berkley, CA) synchronized to MRI data acquisition, on a mylar screen positioned across the scanner bore via an LCD projector. Participants viewed the stimuli via an angled mirror. Prior to the fMRI experiment, participants completed a self-report motor imagery questionnaire (Motor Imagery Questionnaire-Revised Second Version; MIQ-RS)^27,28^ to ensure there were no pre-existing group differences in imagery, and engaged in a familiarization period whereby participants were oriented to the task and type of motor imagery (first person, kinaesthetic) whereby they listened to an auditory script and watched a 30 s clip of the darts task. Participants in the PP-MIP and MIP-PP groups underwent fMRI sessions at three time points (pre/mid/post).

### Behavioural analysis

All statistical analyses were performed using R (R project for statistical computing) with an a priori alpha of 0.05 denoting significance. For the participants in the MIP-PP and PP-MIP groups, separate ANOVAs were conducted to ensure similarity in imagery performance and ability across groups. Specifically, following Shapiro–Wilks and Bartlett’s tests to ensure that data passed the assumptions of ANOVA, MIQ-RS scores were tabulated across participants for each imagery condition (kinaesthetic and visual) and a 2 (imagery condition) X 2 (group) mixed ANOVA was conducted to assess the between group effects of imagery condition on MIQ-RS score, as previously reported^40^. Responses for both manipulation checks during the neuroimaging sessions were averaged across participants for each run and scan to ensure similarity in imagery performance across groups. Separate 3 (time point) X 2 (group) mixed ANOVAs were conducted on each outcome measure (engagement, quality) to assess the between group effects on task engagement and imagery quality.

For all performance related outcomes, following prior work, the first throw of each test block was considered a ‘warmup’ and excluded from statistical analyses. (i.e., leaving 15 total throws per test session). Outliers were identified as throws that exceeded three standard deviations above the mean for each participant across sessions and were removed from further analyses. Our task-related outcomes, RE was calculated for each throw, and consistency (bivariate variable error; BVE)^29–34^ were derived from the digitized data (with the bullseye considered the point of origin (0,0) and error calculated in the X and Y plane from the point of origin). Changes related to both RE and BVE were assessed using two separate linear mixed effects (LME) model conducted using the LME4 package^91^ in assessing RE on the dart throwing task as a function of group, time point, and their interaction with participant entered as a random effect. A model including age and sex was shown to significantly improve the base model, measured using Akaike Information Criterion^92^. Thus, we included terms for age and sex in the final model. Notably, the BVE analysis included one observation per participant per time point, as BVE is calculated across throws within a session (i.e., BVE was first determined for each participant at each test session). To characterise improvements in performance within each group (i.e., between test sessions), we conducted posthoc tests on both mean RE and BVE using Tukey’s HSD in conjunction with effect sizes calculated on both mean RE and BVE. All Tukey’s HSD were conducted using the multcomp package with an a priori significance value of *p* < 0.05, corrected via the single-step method^93^. Importantly, mean RE was also determined for each participant at each test session, to permit the calculation of effect sizes.

### ACF1

The autocorrelation lag-1 (ACF1) is a coefficient of the correlation of two values in time series, and was employed to quantify the distance between two consecutive movements resulting in a measure of the correction factor of throws made on a trial-by-trial basis^35,36,94^. The ACF1 was calculated separately for deviations in the X and Y plane from the point of origin (0,0) using the digitized data, and with the mean correction factor calculated across the two planes. As ACF1 approaches 1, error between endpoints of two consecutive movements decreases. Thus, learning is indicated as the ACF1 approaches 1. Formally, the ACF1 in the X and Y planes are defined as follows:

$$ ACF1_{x} = \frac{{E\left\{ {x\left( n \right)x\left( {n + 1} \right)} \right\}}}{{E\left\{ {x\left( n \right)x\left( n \right)} \right\}}} $$

$$ ACF1_{y} = \frac{{E\left\{ {y\left( n \right)y\left( {n + 1} \right)} \right\}}}{{E\left\{ {y\left( n \right)y\left( n \right)} \right\}}} $$

The ACF1 analysis was applied using customized functions in MATLAB (Mathworks, Inc). For group-level analyses, we computed a mean ACF1 using resultant ACF1 values in the X and Y plane. A 3 (group) X 4 (time point) mixed ANOVA was conducted on the mean ACF1 across participants (using the absolute value, such that deviations in any direction were treated equally), and effect sizes were calculated to characterise training-related improvements in performance for both halves of training, as well as overall (i.e., between day 1 and 5, day 6 and 10, and day 1 and 10, respectively). Posthoc tests were conducted using Tukey’s HSD to examine any effects resulting from the ANOVA.

### Kinematics

Following prior work, initial kinematic variables of interest included shoulder angle, elbow angle, release and preparation time^32,33^, and were derived from video data, analyzed using Dartfish Pro motion analysis software (Dartfish HQ, Fribourg, Switzerland). Joint angles (of the throwing arm) were assessed at two points of the dart throw (i.e., during ‘take back’ or maximum flexion of the elbow, and during the ‘release point’ or the point at which the dart left the participant’s hand), by placing anatomical markers placed on the acromion process, olecranon, and highest point of the iliac crest in line with the coronal plane of the body (for shoulder angle) or styloid process of the throwing arm (for elbow angle). Release time was defined as the time between maximum elbow flexion to the point of release. Preparation time was defined as the time between the point of release to maximum flexion of the subsequent throw. Yet, as little is known about the best kinematics to predict performance on a darts throwing task amongst non-experts, we conducted an exploratory analysis to identify which variables best predicted performance. A linear mixed effects model was conducted using the LME4 package^91^ assessing RE on the darts throwing task as a function of the aforementioned kinematic outcomes (shoulder and elbow angles at maximum flexion and release, preparation and release time) entered as fixed effects, and participant entered as a random effect (Table 4). Shoulder and elbow angles at release in conjunction with shoulder angle at take back were determined to predict accuracy of the darts throw. This analysis is included as Supplementary Material (see Supplementary Table 5). Next, we computed a ‘global kinematic variability’ score, equally weighted across these three variables (using SD calculated across trials per test session) for each participant)^33^,for group-level analyses. We also calculated angular velocity, defined as the elbow angle at maximum flexion subtracted from the point of release and divided by the throwing time, to assess changes in average angular velocity as a function of training at the group-level^33^. Separate 3 (group) × 4 (time point) mixed ANOVAs were conducted on global kinematic variability and angular velocity to assess the between-group effects of training, and effect sizes were calculated to characterise training-related improvements in performance for both halves of training, as well as overall (i.e., between day 1 and 5, day 6 and 10, and day 1 and 10, respectively). Posthoc tests were conducted using Tukey’s HSD to examine any effects resulting from the ANOVAs.

### fMRI preprocessing and analysis

All fMRI data was processed using the Oxford Centre for Functional MRI of the Brain’s (FMRIB) software library (FSL v.5.0.10; FMRIB Oxford, www.fmrib.ox.ac.uk/fsl) and fMRI Expert Analysis Tool (FEAT) version 5.0.10 [part of FSL]^90^). Anatomical data (T1 and T2 scans) was preprocessed using FLIRT and FNIRT to perform registration-based skull-stripping to template brain MNI152_T1_1mm. Functional data was preprocessed (including motion correction with MCFLIRT^37^, field inhomogeneity-induced distortion correction with reverse-phase encoded blips using TOPUP^39^, brain extraction using BET^38^, spatial smoothing using a Gaussian kernel (FWHM 5 mm), and high-pass temporal filtering at 0.01 Hz to remove low-frequency noise) and images were then rigid-body spatially co-registered (using 6DOF) to the processed anatomical image using FLIRT. Further, images were then combined with the non-linear registration to MNI152_1mm with a voxel size of 3 mm for group comparison of individual fMRI results.

Individual statistical activation maps were calculated within each run using a general linear model (GLM) with FEAT, with motion outliers included as confounds, determined from a contrast of imagery (darts) versus rest computed for each run (first-level analysis). A second-level analysis combined all runs in a fixed effects model to produce contrast of parameter estimate (COPE) maps for each individual and time point (pre/mid/post). High-level group analyses were carried out using FLAME (FMRIB's Local Analysis of Mixed Effects) model with FEAT using the averages of the lower-level COPE maps, to assess the impact of training modality on resultant patterns of motor imagery-related brain activity. Specifically, between-group comparisons were conducted at each time point (i.e., pre/mid/post-training). Consistency (BVE) was added as a covariate for the comparisons at the mid- and post-training scan, to account for any differences in performance noted across participants (i.e., such that resultant differences in motor imagery-related brain activation noted across groups are attributable to the modality in which each group trained, vs. the extent to which learning occurred). BVE was then correlated with resultant motor imagery-related activity to determine how resultant brain activity was modulated by the extent to which learning occurred. We adjusted for BVE as consistency is shown to be a more stable measure than accuracy and less influenced by external factors (e.g., time-of-day, fatigue)^31,95,96^. Within-group comparisons for the first and second half of training (i.e., pre- vs. mid-training scan; and mid- vs. post-training scan) were conducted for each group. Of note, between- and within-group comparisons for the pre and mid-training scan are previously reported^40^. All analyses used a corrected cluster threshold of Z > 2.0 and significance threshold of *p* < 0.05, corrected for family-wise error.

## Supplementary information

Supplementary Information 1.
