# Supplementary material for: Leveraging the effector independent nature of motor imagery when it is paired with physical practice
Source: Sci Rep. 2020 Dec 7;10:21335. doi: 10.1038/s41598-020-78120-9 (PMC7721807; doi:10.1038/s41598-020-78120-9)
Supplement: Supplementary file 1 — Supplementary Information 1. [file 41598_2020_78120_MOESM1_ESM.pdf]

LEVERAGING THE EFFECTOR INDEPENDENT NATURE OF MOTOR IMAGERY WHEN IT IS PAIRED WITH PHYSICAL PRACTICE

Sarah N. Kraeutner[1,2], PhD, Jennifer L. McArthur[3], Paul H. Kraeutner[3,], PhD, MASc, David A. Westwood [4,5], PhD, Shaun G. Boe[3-6], PhD, MPT

[1]Brain Behaviour Laboratory, University of British Columbia, Vancouver, British Columbia, Canada V6T1Z3
[2]Department of Physical Therapy, University of British Columbia, Vancouver, British Columbia, Canada V6T1Z3
[3]Laboratory for Brain Recovery and Function, Dalhousie University, Halifax, NS, Canada B3H4R1
[4]Department of Psychology and Neuroscience, Dalhousie University, Halifax Nova Scotia, Canada B3H4R2
[5]School of Health and Human Performance, Dalhousie University, Halifax, Nova Scotia, Canada B3H4R2
[6]School of Physiotherapy, Dalhousie University, Halifax, Nova Scotia, Canada B3H4R2

Address for Correspondence
Dr. S.G. Boe
School of Physiotherapy, Dalhousie University
Rm 407, 4th Floor Forrest Building
5869 University Avenue
PO Box 15000
Halifax, Nova Scotia, Canada, B3H 4R2
Email: s.boe@dal.ca
Tel.: +1-902-494-6360
Fax: +1-902-494-1941

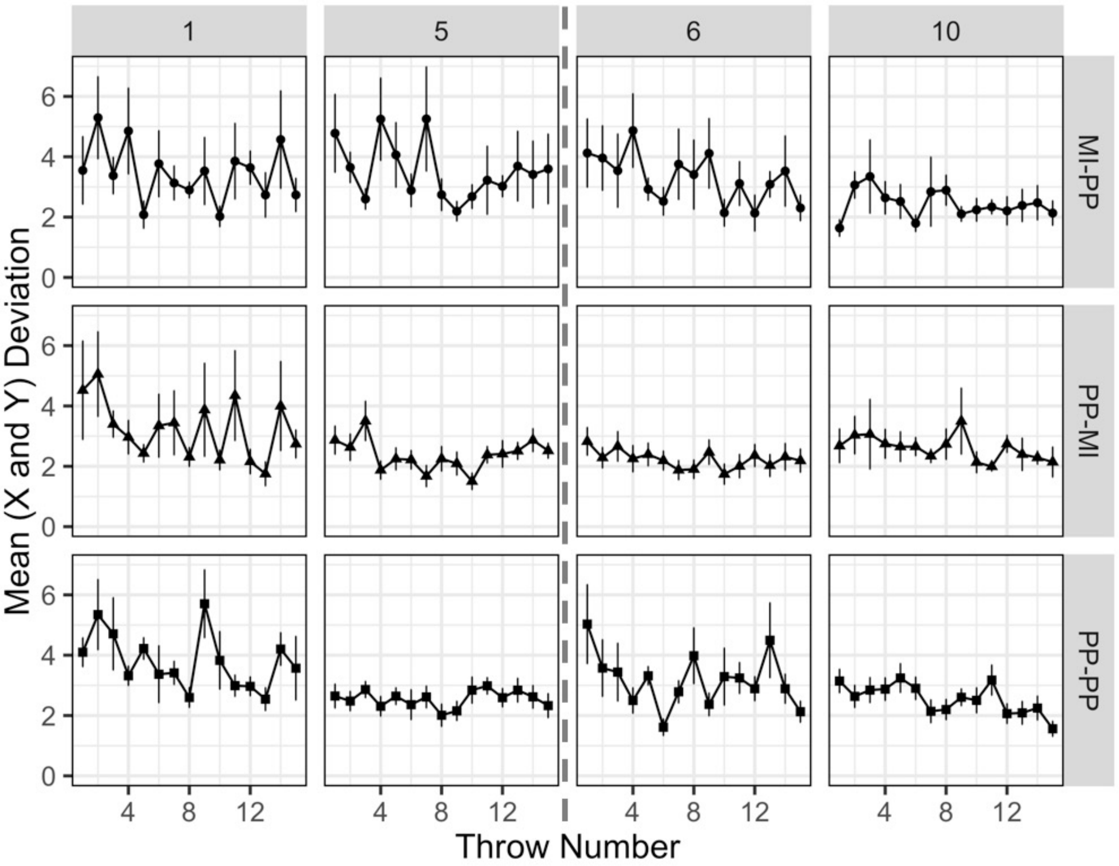

Supplementary Figure 1. Visualization of trial-by-trial corrections. Test sessions (days 1, 5, 6, and 10) are shown across the top as separate panels. The average deviation from the bullseye in the X and Y plane are shown across throws ('Throw Number' shown across the x-axis), for each test session. Error bars depict standard error. As shown via autocorrelation lag-1 (ACF1) calculations and effect sizes within groups (Table 1), changes in ACF1 were only observed in the second half of training (i.e., between days 6 and 10).

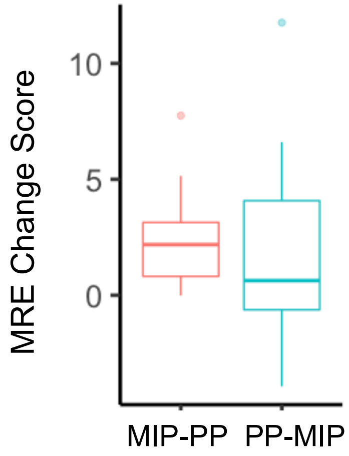

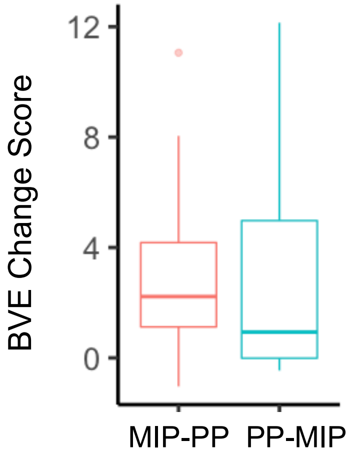

Supplementary Figure 2. Boxplots of changes scores, related to mean radial error (MRE; left) and bivariate variable error (BVE; right), calculated to investigate an additive effect of motor imagery relative to five days of physical practice across mixed-modality groups (i.e., day 1 vs. 10 in the MIP-PP group; day 1 vs. 5 in the PP-MIP group).

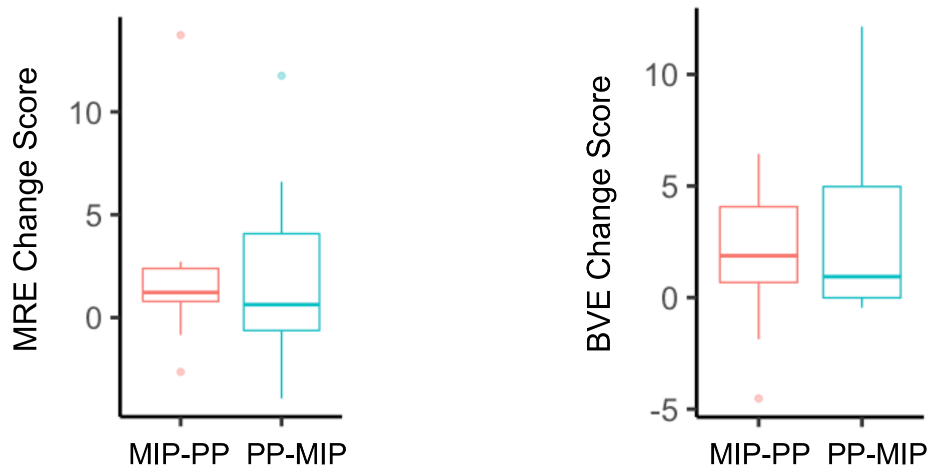

Supplementary Figure 3. Boxplots of changes scores, related to mean radial error (MRE; left) and bivariate variable error (BVE; right), calculated to investigate physical practice-based effects across mixed-modality groups (i.e., day 6 vs. 10 in the MIP-PP group; day 1 vs. 5 in the PP-MIP group).

Supplementary Table 1

MIQ-RS scores and data from imagery manipulation checks conducted during neuroimaging sessions (mean and SD for each group). Data for the pre- and mid-training time points is previously reported in [38]).

		Group	
		MIP-PP	PP-MIP
MIQ-RS score	Kinaesthetic (/49)	35.6 (12.0)	38.7 (8.1)
	Visual (/49)	38.7 (8.3)	39.9 (7.3)
	Time point		
MI engagement (/5)	Pre-training	4.3 (0.7)	4.3 (0.5)
	Mid-training	4.3 (0.7)	4.1 (0.6)
	Post-training	4.4 (0.7)	4.1 (0.7)
MI quality (/5)	Pre-training	4.2 (0.7)	4.0 (0.7)
	Mid-training	4.3 (0.6)	3.9 (0.6)
	Post-training	4.4 (0.5)	4.0 (0.5)

Supplementary Table 2. Results from Tukey's HSD posthoc tests examining differences between each time point for each group, performed using the 'multcomp' package in R, with p values corrected using the single-step method.

Radial Error		Day 1	Day 5	Day 6	Day 10
MIP-PP	Day 1	-	0.998	0.963	0.003*
	Day 5	-	-	0.849	<0.001*
	Day 6	-	-	-	0.013*
	Day 10	-	-	-	-
PP-MIP	Day 1	-	0.001*	<0.001*	0.050
	Day 5	-	-	0.943	0.648
	Day 6	-	-	-	0.310
	Day 10	-	-	-	-
PP-PP	Day 1	-	<0.001*	0.012*	<0.001*
	Day 5	-	-	0.009*	0.996
	Day 6	-	-	-	0.019*
	Day 10	-	-	-	-

Bivariate Variable Error		Day 1	Day 5	Day 6	Day 10
MIP-PP	Day 1	-	0.703	0.535	0.013*
	Day 5	-	-	0.993	0.204
	Day 6	-	-	-	0.327
	Day 10	-	-	-	-
PP-MIP	Day 1	-	0.005*	0.010*	0.122
	Day 5	-	-	0.995	0.660
	Day 6	-	-	-	0.803
	Day 10	-	-	-	-
PP-PP	Day 1	-	<0.001*	0.748	<0.001*
	Day 5	-	-	0.009*	0.994
	Day 6	-	-	-	0.020*
	Day 10	-	-	-	-

Note: *indicates significance ($p < 0.05$)

Supplementary Table 3. Results from Tukey's HSD posthoc tests examining the main effect of time point observed for global kinematic variability, angular velocity, and ACF1, with p values corrected using the single-step method.

		Day 1	Day 5	Day 6	Day 10
Global Kinematic Variability	Day 1	-	0.008*	0.731	0.007*
	Day 5	-	-	0.121	1.00
	Day 6	-	-	-	0.109
	Day 10	-	-	-	-
Angular Velocity	Day 1	-	0.428	0.456	0.353
	Day 5	-	-	1.00	1.00
	Day 6	-	-	-	1.00
	Day 10	-	-	-	-
ACF1	Day 1	-	0.993	0.993	0.842
	Day 5	-	-	1.00	0.690
	Day 6	-	-	-	0.687
	Day 10	-	-	-	-

Note: *indicates significance ($p < 0.05$)

Supplementary Table 4

MNI coordinates of the peak maxima resulting from comparisons conducted to assess within-group differences in motor imagery-related brain activation following training of the dart throwing task.

Anatomical region		MNI coordinates (mm)			Z-score
		x	y	z	
PP-MIP group					
Mid > pre-training	L Middle temporal gyrus	-66	-14	-4	4.51
	L Thalamus	-4	-20	-7	4.38
	L Anterior cingulate	-14	49	-3	3.54
	L Middle frontal gyrus (orbital)	-18	57	-16	3.53
	L Medial orbitofrontal cortex	-10	56	-1	3.36
	R Supramarginal gyrus	44	-39	22	4.53
	R Medial frontal gyrus	6	38	54	3.65
	R Medial orbitofrontal cortex	9	59	0	3.51
Pre > mid-training	-	-	-	-	-
Post </> mid-training	-	-	-	-	-
MIP-PP group					
Mid </> pre-training	-	-	-	-	-
Post </> mid-training	-	-	-	-	-

Note: instances in which no significant differences in activation were noted are indicated by dashed lines.

Supplementary Table 5

Linear mixed effects conducted on kinematic outcomes to determine variables linked to radial error on the dart throwing task.

	**Radial error (cm)**		
Predictors	*Estimates*	*CI*	*p*
(Intercept)	7.53	6.67 – 8.38	**<0.001**
Elbow angle at release	0.82	-0.11 – 1.76	0.085†
Elbow angle at take back	-0.29	-0.83 – 0.25	0.291
Shoulder angle at release	-0.68	-1.23 – -0.13	**0.015**†
Shoulder angle at take back	-0.69	-1.26 – -0.12	**0.018**†
Release time	-0.27	-1.10 – 0.57	0.530
Angular velocity	-0.57	-1.55 – 0.42	0.262
**Random Effects**			
σ^2	31.39		
$\tau_{00\ \text{participant}}$	5.89		
ICC $_{\text{participant}}$	0.16		
Observations	1840		
Marginal R^2 / Conditional R^2	0.034 / 0.187		

†Note: Stemming from these results, shoulder angle at both release and take back (statistically significant), as well as elbow angle at release (trending) were included in further analyses of global kinematic variability.

Supplementary Table 6.

Change scores related to MRE and BVE are shown for each group (i.e., day 6 vs. 10 in PP-MIP group and PP-PP group; day 1 vs. 5 in the MIP-PP group), calculated to probe the existence of an additive effect of five days of motor imagery and five days of physical practice, relative to five days of physical practice. Positive numbers are indicative of a greater improvement.

	MRE change score *(SD)*	BVE change score *(SD)*
MIP-PP (day 1 minus 10)	2.47 (2.32)	3.14 *(3.63)*
PP-MIP (day 1 minus 5)	2.08 *(4.37)*	3.47 *(4.75)*
PP-PP (day 1 minus 5)	3.17 *(2.30)*	3.55 *(3.24)*

Supplementary Table 7.

Change scores related to MRE and BVE are shown for each group (i.e., day 6 vs. 10 in PP-MIP group and PP-PP group; day 1 vs. 5 in the MIP-PP group), calculated to demonstrate physical practice-based effects for each group. Positive numbers are indicative of greater improvement.

	MRE change score *(SD)*	BVE change score *(SD)*
MIP-PP (day 5 minus 1)	2.13 (4.15)	1.75 *(3.11)*
PP-MIP (day 10 minus 6)	2.08 *(4.37)*	3.47 *(4.75)*
PP-PP (day 10 minus 6)	3.17 *(2.30)*	3.55 *(3.24)*
